# Control of *Fusarium verticillioides* (Sacc.) Nirenberg and Fumonisins by Using a Combination of Crop Protection Products and Fertilization

**DOI:** 10.3390/toxins10020067

**Published:** 2018-02-02

**Authors:** Richard Raphael Madege, Kris Audenaert, Martin Kimanya, Bendantukuka Tiisekwa, Bruno De Meulenaer, Boris Bekaert, Sofie Landschoot, Geert Haesaert

**Affiliations:** 1 College of Agriculture, Sokoine University of Agriculture, P.O. Box 3005, Morogoro, Tanzania; richard.madege@ugent.be (R.R.M.); tiisekwa@suanet.ac.tz (B.T.); 2 Department of Plants and Crops, Faculty of Bioscience Engineering, Ghent University, Valentin Vaerwyckweg 1, BE-9000 Ghent, Belgium; kris.audenaert@ugent.be (K.A.); boris.bekaert@ugent.be (B.B.); 3 School of life Sciences and Bio Engineering, The Nelson Mandela African Institution of Science and Technologies, P.O. Box 447, Arusha, Tanzania; martin.kimanya@nm-aist.ac.tz; 4 Department of Food Technology, Safety and Health, Faculty of Bioscience Engineering, Ghent University, Coupure Links 653, BE-9000 Ghent, Belgium; bruno.demeulenaer@ugent.be; 5 Department of Data Analysis and Mathematical Modelling, Faculty of Bioscience Engineering, Ghent University, Valentin Vaerwyckweg 1, BE-9000 Ghent, Belgium; sofie.landschoot@ugent.be

**Keywords:** endosulfan, ear rot, fertilizer, fumonisin, fungicide, insecticide, triadimenol + tebuconazole

## Abstract

*Fusarium verticillioides* is the most common fungal pathogen associated with maize ear rot in Tanzania. In a two-year trial, we investigated the efficacy of crop protection (insecticide and/or fungicide) and fertilizer (nitrogen and/or phosphorus) treatments in reducing the occurrence of *F. verticillioides* and its mycotoxins in maize grown in Tanzania. Seasonal differences were seen to have a substantial influence on the incidence and severity of insect infestation, *Fusarium* ear and kernel rot, biomass of *F. verticillioides* and contamination with fumonisins. With regard to the application of fertilizers, it was concluded that the impact on maize stalk borer injury, *Fusarium* symptoms and fumonisin levels was not significant, whereas crop protection significantly reduced maize damage. The application of an insecticide was most effective in reducing insect injury and as a result of the reduced insect injury the insecticide treatment also resulted in a significant decrease in *Fusarium* symptoms. In 2014, fumonisin levels were also significantly lower in maize treated with an insecticide. Additionally, significant positive correlations between insect damage and *Fusarium* symptoms were observed. In conclusion, this study clearly shows that application of an insecticide alone or in combination with a fungicide at anthesis significantly reduces insect damage and consequently reduces *F. verticillioides* infection and associated fumonisin contamination.

## 1. Introduction

Maize (*Zea mays* L.) is a cereal crop grown worldwide in a range of agro-ecological environments. It is the major staple food [1], feed compound [2], and industrial raw material [3] used throughout the world. In Tanzania, maize is produced in all 26 regions, predominantly by smallholder farms. Maize is grown on approximately 4.12 million hectares and provides 60% of dietary calories and more than 35% of utilizable protein to the Tanzanian population [4]. It is also a major source of income for the majority of smallholders, since about 40% of the production is sold on the local markets particularly around the urban centers. The annual consumption per capita is 73 kg per year. Therefore, the quality of maize consumed significantly contributes to the livelihood of the majority of the Tanzanians [5]. Unfortunately, up to 40% of the maize yield is lost, mainly due to pre-harvest attacks by insects and fungal pathogens [6]. Among the deleterious pathogens are species of the genus *Fusarium* [7,8]. *Fusarium* infection does not only cause losses in quality and quantity of maize yield, but also contaminates the crop with secondary toxic metabolites called mycotoxins. Mycotoxins are of major concern because they pose health problems in humans and animals [9]. Studies show that occurrence and exposure to mycotoxins in Tanzania is high due to high pre- and post-harvest contamination. Moreover, the African diet is mainly composed of maize which results in a high exposure level [10,11,12,13]. The weather conditions from the reproductive stage until kernel drying seem to be critical for infection with *Fusarium verticillioides* and subsequent contamination with fumonisins [14]. Furthermore, *Fusarium verticillioides* has a dual nature and can be present as both a pathogen and a symptomless endophyte which penetrates the plant through the roots [15]. Due to this, the pathogen can be latently present, ready to attack whenever the maize physiology changes.

Good agricultural practices (GAP), which mitigate biotic and abiotic stressors, can reduce mycotoxin contamination significantly [16]. GAP include soil and crop residue management, growing tolerant varieties, crop rotation, fertilization, insect management, irrigation, and timely planting [17]. Planting Bt maize, genetically engineered maize which expresses proteins from *B. thuringiensis* that are poisonous to certain insect pests, is an effective control strategy for maize stem borer resulting in a reduced *F. verticillioides* infection [18] and reduced fumonisin production and accumulation [19,20,21]. However, bearing the endophytic nature of *F. verticillioides*, the use of Bt maize can only be effective in controlling infection which occurs through insect damage. Apart from being uncommon to Tanzanian smallholder farmers, the report that maize stem borer has developed resistance to Bt maize [22] is another setback. In Tanzania, studies related to agrochemicals have traditionally been carried out mainly during vegetative growth phase to improve yield of maize [23,24]. Unfortunately, associations of these treatments with contamination of fumonisins and other mycotoxins are lacking in Tanzania. The aim of this study was to evaluate the effect of fertilizer (phosphorus and nitrogen), and crop protection (endosulfan and triadimenol + tebuconazole) on the occurrence of *F. verticillioides* and its mycotoxins fumonisin B1 and fumonisin B2 in maize. The field experiments were carried out under Tanzanian growing conditions.

## 2. Results

### 2.1. Influence of Growing Season on Insect and Fungal Incidence

The weather data recorded in the experimental area during the maize growing seasons 2013 and 2014 are presented in Table 1. While the average temperature during both growing seasons was similar, 24 °C in 2013 and 25 °C in 2014, the months of March, April, May and June during 2014 were on average more humid (64%) than in 2013 (56%). During the same cropping period, the area received more rainfall in 2013 (607 mm) than in 2014 (551 mm). The growing season of 2014 received less rain during the early months (March and April) of the season and more rain during the second part of the season (May and June), the period when the crop goes from anthesis towards maturity.

Since weather conditions are important factors influencing insect and fungal development, it was assessed whether there were significant differences in entomological, mycological and fumonisin contamination between seasons. It was concluded that the proportion of kernel injury and fumonisin contamination were significantly higher in 2013 compared to 2014. In contrast, *Fusarium* ear rot was lower in 2013 compared to 2014 and maize kernels from 2013 contained a lower biomass of *F. verticillioides* (Table 2).

### 2.2. Effect of Fertilization and Crop Protection on Maize Stalk Borer (MSB) and Fusarium Symptoms

**Figure 1:**
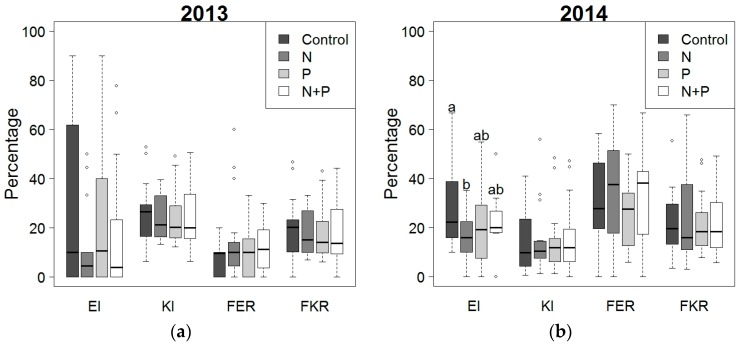
Effect of fertilization (unfertilized control, nitrogen (N), phosphorus (P) and N + P) on the percentage of maize stalk borer ear injury (EI), kernel injury (KI), *Fusarium* ear rot (FER) and *Fusarium* kernel rot (FKR) during 2013 (**a**) and 2014 (**b**). No significant effects of fertilizer were noted, except for 2014 for EI the difference between the control and N was significant according to a Dunnett T3 test.

The two-way ANOVA revealed that for the variables ear injury, kernel injury, *Fusarium* ear rot and *Fusarium* kernel rot, there was no significant interaction between the factors fertilization and crop protection (*p*-value > 0.05); as a result, the effect of both factors were analyzed separately. In Figure 1 the influence of fertilization on the incidence of ear and kernel injury due to MSB and the incidence of *Fusarium* ear and kernel rot due to *Fusarium* is displayed. Concerning the effect of fertilizer, it was seen that in both 2013 and 2014, there was no significant impact of fertilizer application on the symptoms of both MSB (ear and kernel injury) and *Fusarium* (*Fusarium* ear and kernel rot). Except in 2014, the fields fertilized with N showed a significantly lower ear injury compared to the unfertilized control fields.

**Figure 2:**
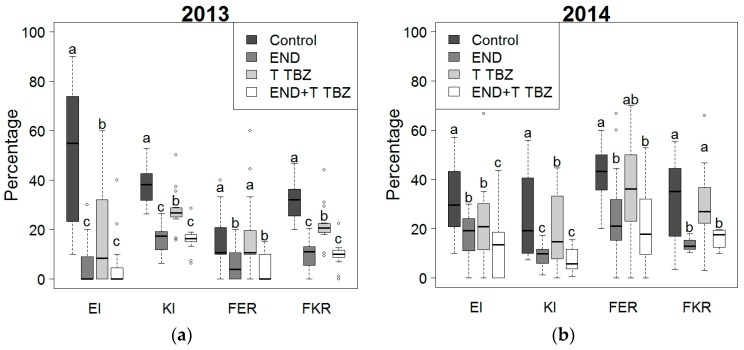
Effect of crop protection (control, endosulfan (END), triadimenol + tebuconazole (T TBZ) and endosulfan combined with triadimenol + tebuconazole (END + T TBZ)) on percentage of maize stalk borer ear injury (EI), kernel injury (KI), *Fusarium* ear rot (FER) and *Fusarium* kernel rot (FKR) during 2013 (**a**) and 2014 (**b**). Different letters indicated significant differences between treatments according to Dunnett T3 test.

With regard to the impact of crop protection, Figure 2 shows significant differences (*p*-value < 0.05) between treatments in their efficacy towards insect damage (ear and kernel injury) and *Fusarium* symptoms (*Fusarium* ear and kernel rot). Maize from plots that were not treated with crop protection products had, on average, the highest incidences of insect damage and *Fusarium* symptoms. In 2013, the untreated plots were always significantly more affected than the plots treated with insecticides and/or fungicides (except for *Fusarium* ear rot). In 2014, the difference in *Fusarium* incidence (*Fusarium* ear and kernel rot) between the control and the fungicide triadimenol + tebuconazole was not significant. Maize plots treated with endosulfan or the combination of endosulfan and triadimenol + tebuconazole were always significantly less affected than untreated control plots. When comparing the endosulfan treatment with the combined application of endosulfan and triadimenol + tebuconazole, only for ear injury in 2014 significant differences between both treatments were observed, where the combination of the insecticide and fungicide resulted in significantly lower ear injury compared to the application of only a fungicide or insecticide.

### 2.3. Effect of Fertilization and Crop Protection on Fumonisins

**Figure 3:**
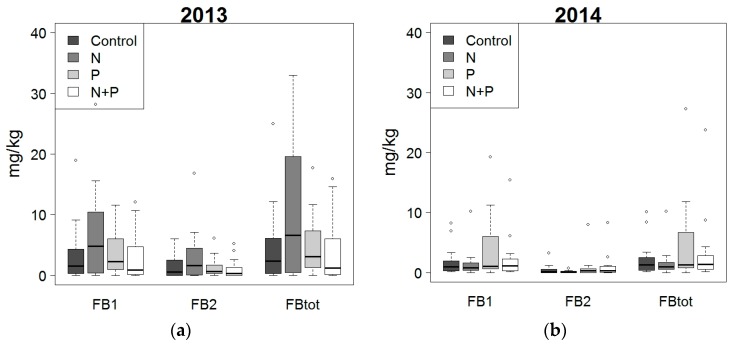
Effect of fertilization (control, nitrogen (N), phosphorus (P) and N + P) on the level of fumonisin B1 (FB1), fumonisin B2 (FB2) and the total fumonisin level (FBtot) in 2013 (**a**) and 2014 (**b**). No significant effect of fertilizer was noted.

**Figure 4:**
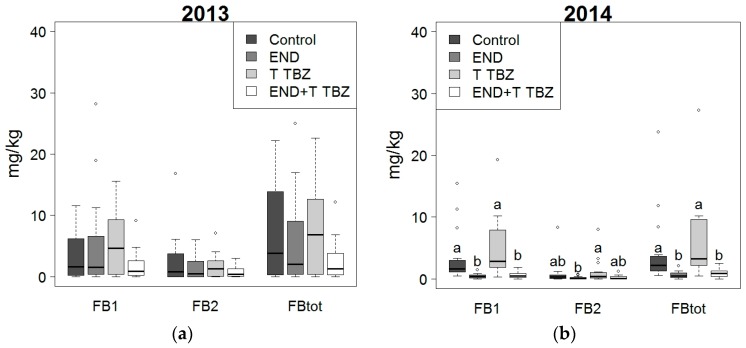
Effect of crop protection (control, endosulfan (END), triadimenol + tebuconazole (T TBZ) and endosulfan combined with triadimenol + tebuconazole (END + T TBZ)) on the level of fumonisin B1 (FB1), fumonisin B2 (FB2) and the total fumonisin level (FBtot) in 2013 (**a**) and 2014 (**b**). Different letters indicated significant differences between treatments according to Dunnett T3 test.

Secondly, the influence of fertilization and crop protection on the accumulation of FB1, FB2 and the sum of FB1 and FB2 (FBtot) was evaluated (Figure 3 and Figure 4). According to the two-way ANOVA, there was no significant interaction between fertilization and crop protection (*p*-value > 0.05) with respect to toxin contamination. The application of fertilizers did not have a significant effect (*p*-value > 0.05) on the fumonisin levels. Concerning the effect of crop protection, in 2013 no significant (*p*-value > 0.05) differences in fumonisin levels were observed between the various treatments. With reference to the untreated control, in 2014 a significant reduction in fumonisins was realized thanks to the treatment with endosulfan and the combined treatment with endosulfan and triadimenol + tebuconazole. Furthermore, it can be seen that in both 2013 and 2014, the fumonisin levels in maize from the plots treated with triadimenol + tebuconazole were higher than in the untreated control plots, however, this difference was not significant.

Additionally, it was concluded that 55% of the samples from 2013 exceeded the threshold of 2 mg/kg (Commission Regulation 1881/2006 for unprocessed maize). Although, based on the data from 2013, the effects of crop protection or fertilization on fumonisin levels were not significant, it was seen that for the treatments endosulfan and triadimenol + tebuconazole in combination with N fertilization or N + P fertilization, and the treatment with endosulfan in combination with P + N fertilization only one out of the four replicates exceeded the threshold of 2 mg/kg. In 2014, 35% of the samples exceeded the legal threshold, and for the treatments mentioned above, none of the samples exceeded the threshold of 2 mg/kg.

### 2.4. Effect of Fertilization and Crop Protection on the Biomass of F. verticillioides

**Figure 5:**
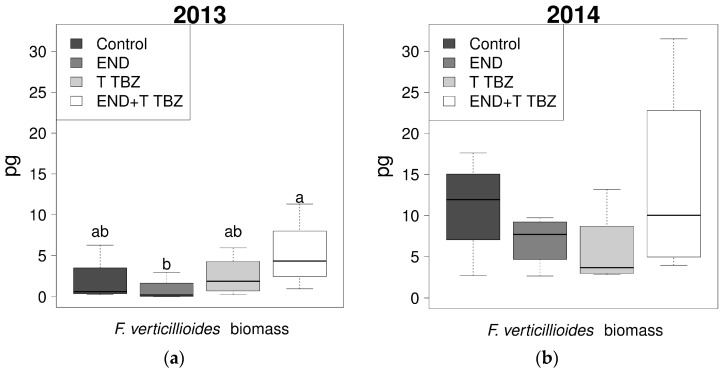
Effects of endosulfan (ES), triadimenol + tebuconazole (T TBZ), the combination of endosulfan and triadimenol + tebuconazole (END + T TBZ) and no chemical treatments (Control) on the *F. verticillioides* biomass in maize kernels (pg DNA per gram maize kernels) of the 2013 (**a**) and 2014 (**b**) cropping seasons. Different letters indicated significant differences between treatments according to Dunnett T3 test.

The biomass of *F. verticillioides* varied significantly (*p*-value < 0.05) between years, with a significantly higher colonization in 2014 (Figure 5). Significant differences (*p*-value < 0.05) were established concerning the efficacy of endosulfan and triadimenol + tebuconazole in 2013. In 2013, the DNA biomass of the *F. verticillioides* in maize grains treated with endosulfan was significantly lower compared to the biomass of the maize grains treated with both endosulfan and triadimenol + tebuconazole. In 2014, no significant differences between treatments were observed, however, remarkably, similar to 2013, the combination of endosulfan and triadimenol + tebuconazole resulted in the highest *F. verticillioides* biomass.

### 2.5. Correlation between Variables

**Figure 6:**
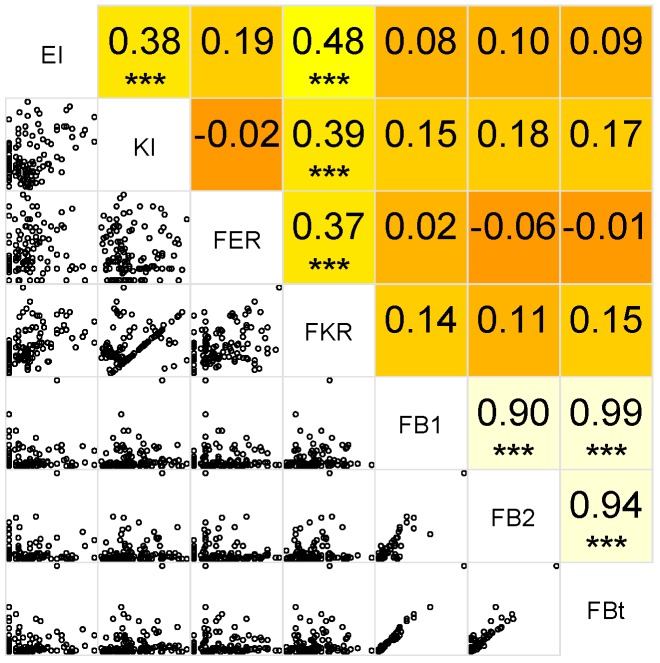
Correlation between maize stalk borer injury ear and kernel injury (EI and KI), *Fusarium* ear and kernel rot (FER and FKR), and fumonisin B1, fumonisin B2, and the total fumonisin level (FBt). Significant correlations at α = 0.05 are indicated with ***.

In Figure 6, the correlations between maize stalk borer ear and kernel injury, *Fusarium* ear and kernel rot, and fumonisin B1 (FB1), B2 (FB2), and the total fumonisin level (FBt) are displayed. It can be seen that ear and kernel injury on the one hand and *Fusarium* ear and kernel rot on the other hand are significantly positively correlated. Furthermore, there was a significant positive correlation between *Fusarium* kernel rot and ear and kernel injury. In contrast, *Fusarium* ear rot showed no significant correlation with MSB injury. Additionally, FB1, FB2 and total amount of fumonisins were highly positively correlated. Concerning the correlations between fumonisin levels and MSB or *Fusarium* symptoms, highest positive correlations were observed between kernel injury and FB2 (0.18) and the total amount of fumonisins (0.17), followed by the correlation between *Fusarium* kernel injury and fumonisin (0.15), however, these correlations were not significant.

## 3. Discussion

This two-year field experiment revealed that, even in a stable tropical climate, *Fusarium* ear rot and fumonisin contamination can vary substantially between seasons. Significant differences in *Fusarium* biomass and mycotoxins across cropping cycles were observed. The occurrence of fumonisins in maize kernels was higher in 2013 than in 2014. These differences can be attributed to differences in weather conditions between both growing seasons. The year 2013 had a higher total cumulative rainfall yet the number of rainy days during the cropping season was less than that of 2014. This implies that there were more dry days and hours, suggesting that production of fumonisins was favored by warm, dry weather during the grain-filling period as previously reported in the literature [25]. Parson and Munkvold [18] noted that drought or moisture stress is associated with fumonisin contamination. According to Cao et al. [26], germination of micro and macro conidia of *F. verticillioides* and subsequent infection in maize increases during dry conditions especially at flowering which renders the crop more vulnerable to *F. verticillioides* infection at harvest, perhaps by causing hydric stress in maize plants rendering them more susceptible to insect and fungal attack. An alternative explanation to increased infection of *F. verticillioides* under dry weather at flowering of maize, could be the favorable movement of insects inside the ear and enable fungal infection through spore sticking on insects or the wounds created by the insect tunneling on maize tissues [18]. Dry warm weather during grain filling has an influence on the water activity and temperature which play a key role in modulating fumonisin production. The effects of these factors on fumonisin biosynthesis by *F. verticillioides* and *F. proliferatum* have been largely studied in vitro on maize grains. Higher water availability generally results in higher fumonisin production and higher fungal growth [27]. Optimal conditions for fumonisin production are 0.97–0.98 a_w_ and at temperatures between 20 °C and 30 °C for *F. verticillioides* and between 15 °C and 25 °C for *F. proliferatum*. However, previous studies [27,28] show that, at temperatures that are not optimum for fungal biomass accumulation, fumonisin production in relation to fungal growth may be greater at lower a_w_ values, suggesting that a_w_ stress may enhance fumonisin production. This implies that, the weather conditions (temperature, rainfall and relative humidity) play a decisive role in fungal colonization. Therefore, presence or absence of mycotoxins could largely be a result of the interaction of molds with environmental factors and genetic characteristic of the host which are determinants of disease progress as well as production of mycotoxins [29].

The experiment also showed that fertilization had no significant effect on MSB injury, *Fusarium* ear and kernel rot and fumonisin levels. The low level of success of fertilizer in reducing susceptibility of maize to damage of MSB as observed in this study could be associated with soil nutrient imbalance between organic carbon and synthetic fertilizer whereby high nitrogen rates could alter the biochemical composition of the inner tissues. This explanation is in agreement with a previous report by Altieri and Nicholls [30], who suggested that cultural methods like application of nitrogen fertilizers can affect susceptibility of plants to insect pests by altering plant tissue nutrient levels. Furthermore, despite being one of the most important nutrients for plant growth and disease development, the effects of nitrogen on disease development are inconsistent and contradict each other, and the real causes of this inconsistency are still poorly understood by both plant physiologists and pathologists [31]. While physiologists describe the role of nitrogen as nutritional supplement to plants defense competencies against pathogens, pathologists establish that there is a positive correlation between increment in soil nitrogen and virulence of various pathogens including fungi [32,33].

Indeed, some researchers report that there is a possibility of reduced susceptibility of maize plants to insect pests due to differences in plant health resulting from soil fertility management [34]. Plants with adequate fertilization are likely to have less abiotic stress and less vulnerable to *Fusarium*. Plants suffering from abiotic stress are characterized by lower crop yield and quality, prone to fungal infection and in some cases by higher amounts of mycotoxins [35]. While others report that plant susceptibility to insect pests increased with nitrogen supply in a dose dependent manner [36]. Additionally, it has been reported that nitrogen can increase contamination with fumonisins, since oversupply of nitrogen can potentially increase virulence of a pathogen as it becomes toxic to the plant [37]. Alternatively, the delayed physiological maturity due to nitrogen supplementation might have given longer colonization time of the fungi [38].

This research has demonstrated that antifungal and insecticide application during flowering and grain filling, when the crop is most vulnerable, was effective in reducing MSB infestation. Endosulfan treatment resulted into a reduced proportion of wounded maize kernels due to tunneling of secondary larvae of MSB. Furthermore, the data in this research have shown that controlling MSB with endosulfan also reduced *Fusarium* ear and kernel rot confirming the previous study of Blandino et al. [39]. Furthermore, the fumonisin levels in maize were greatly reduced by the application of endosulfan This two-year experiment has confirmed that spraying endosulfan at silking stage with repetition after 21 days when the crop is at the milk stage is an effective strategy to reduce incidences of cob injuries due to larvae tunneling. This finding conforms to the results of previous studies on the efficacy of insecticide treatments on *Ostrinia nubilalis* (Hübner) and their influence on the mycotoxin contamination of maize kernels [40,41]. The effect of insecticide treatment on reduction of fumonisins and *Fusarium* symptoms is indirect through the reduction of insect vectors and the number of maize kernels which are wounded by the second-generation caterpillar of maize stalk borer (*Busseola fusca*, Fuller). The correlation analysis indeed revealed that there was a significant positive correlation between MSB injury and *Fusarium* kernel rot. The results which link reduction in insect damage on cob and kernels with reduction in ear and kernel rot compares well with effects on the same due to the use of genetically engineered Bt maize [19,42,43]. Furthermore, the observed reduction in fumonisins can be linked to the reduction in cob and kernel injury with subsequent reduction in ear and kernel rot.

Previously, it has been demonstrated that fumonisin levels are positively correlated to the tunnels in ears [39,44], however, in this study no significant correlation between toxins and maize damage was found. Insignificant correlations confirm the previous reports that, mycelial growth and mycelial biomass in maize grains does not necessarily mean production of fumonisins. This is because the conditions which modulate biosynthesis of fumonisins are different from the conditions favorable for growth of fumonisin producing *Fusarium* spp. Samapundo et al. [27] observed that at lower water activity, the optimum temperature for FB production was between 15 °C and 25 °C, while poorest fumonisin production was noted at 30 °C optimum temperature for growth of *F. verticillioides* and *F. proliferatum*.

The results of this research have established that compared to untreated control, the application of triadimenol + tebuconazole generally increased FB contamination in 2014. A recent study [45] established that application of fungicide caused inhibitory effects to growth and morphological development of some structures involved in penetration and colonization of maize grains. Showing agreement with current research, the in vitro experiment by Miguel et al. [45] established that FB1 levels were higher in the presence of fungicide. In addition, Audenaert et al. [46] showed that sub-optimal fungicide concentrations can lead to increased toxin production by *Fusarium* species. Furthermore, a decreased sensitivity of *Fusarium* species towards triazole fungicides has also been observed [47]. Whether fungicides play a direct role as a precursor for fumonisin synthesis remains unfounded although upon degradation it interacts with other elements [48].

From this study it was concluded that the growing season, varying weather conditions, significantly influences pest infestation, infection of *F. verticillioides*, biomass of *F. verticillioides* and contamination with fumonisins. However, it was seen that appropriate management practices can significantly reduce insect injury, *Fusarium* symptoms and toxin levels. Although the effect of fertilization was not significant, no fertilization leads to higher insect infestation and more *Fusarium* symptoms. Controlling insect injury by applying the insecticide endosulfan alone or in combination with the fungicide triadimenol + tebuconazole is the most effective to grow a healthy crop.

## 4. Materials and Methods

### 4.1. Description of the Sampling Site

A field experiment was conducted from March to July during 2013 and 2014 at the experimental fields (6.85° S, 37.65° E, <500 m above sea level) of the Sokoine University of Agriculture, Tanzania. Daily weather data for both growing seasons, including precipitation (mm), air temperature and relative humidity were collected using sensors mounted onto automated data loggers (Umwelt-Geräte-Technik GmbH, Müncheberg, Germany) installed at the experimental site.

### 4.2. Experimental Design

The experimental design of the field trails was always a completely randomized block with four repetitions. Plot size was 4 m × 4 m. The experiment was laid out as a bi-factorial set-up with crop protection products as one and fertilizers as second factor. Table 3 gives an overview of the treatments.

Untreated plots for crop protection products as well as fertilizers were used as control. Phosphorus was applied by banding at 8 cm to 10 cm depth of sowing furrow. Nitrogen was applied in two vegetative stages; when 50% of plants had unfolded the 8th leaf (15 days after planting) and at 50% tasseling (42 days after planting) by banding at about 2–5 cm away from the plants a drill which was created manually by hand. Endosalfan and triadimenol + tebuconazole were applied twice (21 days interval) at silking stage and when kernels have accumulated half of their total dry weight. Each application was as per respective manufacturers’ recommendation. Application was done by spraying directly onto the silk channels using hand driven 15 L knapsack sprayer fitted with HCX Hollow Cone 80° nozzle. Twenty ml of Thionex and 5 mL Rustal were mixed in 15 L water. Seeds of STUKA M1 maize variety were sown at 30 cm intra row spacing and 75 cm inter row spacing. All non-experimental crop husbandry practices were maintained at optimal recommendations.

### 4.3. Entomological and Mycological Observations

At physiological maturity, 30 plants in the middle of each plot were harvested by detaching the cobs for laboratory observation. In the laboratory, cobs were separated from stems followed by removing husks and silks. Cobs were evaluated for ear injury (EI) and *Fusarium* ear rot (FER). EI due to maize stalk borer (MSB) was rated based on a previously established scale ranging from 1 to 7 [39] whereby 1 refers to no injuries, while 7 is more than 60% injury. Disease rating scale of 1 = no visual symptoms and 9 = totally rotted ears without any healthy kernels [49] was used to evaluate FER. After ear evaluation, the kernels were removed from the cobs by hand. A subsample of 1000 seed weight (W1) was determined before sorting out kernels which were symptomatic of insect injury and reweight (W2). Proportion of kernel injury (KI) and *Fusarium* kernel rot (FKR) was expressed as W2/W1 × 100. From each plot, a subsample of 250 g was oven dried at 70 °C for 72 h to determine the sample moisture content and stop any further microbial activity after harvest. The oven dry samples were kept at −20 °C until required for chemical analysis.

### 4.4. Quantification of F. verticillioides Biomass and Fumonisins

Fumonisin B (FB1) and B2 (FB2) in maize were determined using chromatographic method based on Syndenham et al. [50] with slight modifications made by Samapundo et al. [51]. Sample preparation and HPLC analysis were performed as described by Kamala et al. [52]. The extraction of FBs from ground maize sample (15 g) was done by mixing 40 mL of methanol:water (3:1, *v*/*v*) in a 100 mL glass bottle fitted on a laboratory shaker for 1 h. Whatman paper number 4 was used to filter the slurry and the bottle was rinsed with 10 mL of a mixture of methanol:water. The 10 mL of extract was applied to a solid ion exchange (SAX) cartridge (Varian Bond-Elut LRC 500 mg 10 mL, Varian Belgium NV/SA, Sint-Katelyne-Waver, Belgium). The SAX cartridge was conditioned with 5 mL of methanol followed by 5 mL of a methanol:water mix (3:1, *v*/*v*) before applying the extract. The SAX cartridge was washed with 8 mL of methanol:water mix (3:1, *v*/*v*) followed by 3 mL methanol after application of the extract. The elution of FBs from the cartridge was performed with 10 ml of 1% (*v*/*v*) glacial acetic acid in methanol; the eluate was collected followed with evaporation to dryness at 60 °C under a gentle stream of nitrogen using a nitrogen evaporator (Pierce model 18780, ReactiVap, Rockford, IL, USA). The dried FBs were dissolved in 200 μL of methanol and thoroughly mixed with 200 μL of derivatising reagent. This derivatising reagent was prepared by dissolving 40 mg of OPA in a mixture of 1 mL of methanol, 5 mL of 0.1 M sodium tetraborate and 50 μL of β-mercaptoethanol. A total of 20 μL of the mixture were injected into the HPLC for analysis within 8 min. A Shimadzu HPLC system (Shimadzu, Tokyo, Japan) consisting of a Shimadzu LC 20AD pump and fluorescence detector model RF-10AXL was used. Chromatographic separations were performed on stainless steel, Waters Spherisorb^®^ (Milford, MA, USA) ODS-1 5 μm, 4.6 × 200 mm). The mobile phase used was methanol 0.1 M sodium dihydrogen phosphate (75:25, *v*/*v*) mixture adjusted to pH 3.35 with orthophosphoric acid then filtered under vacuum with 0.45 μm filter paper. The mobile phase was set at flow rate of 1 mL/min, and fluorescence of the FB OPA derivatives was detected at wavelengths of 335 nm (excitation) and 400 nm (emission). The LOD of the analytical method was 0.053 mg/kg for FB1 and 0.047 mg/kg for FB2. To evaluate suitability of the method, blank samples of maize flour were spiked with FB1 and FB2 each at 0.1, 0.2, 0.3, 0.4 and 0.5 mg/kg. Spiked maize flour was obtained from healthy kernels sorted from different real samples that had been previously tested and did not contain traces of contamination. No significant differences were observed for percentage recovery obtained at different concentration levels. An *F*-test at a 95% confidence level was used to compare RSD values of recoveries for different concentration levels; the calculated value was found to be less than the critical value. Average recovery values were 106% (15 samples, RSD = 16.6%) and 92% (15 samples; RSD = 15.3%) for FB1 and FB2, respectively.

For quantification of *Fusarium* biomass, DNA extraction was done by following ZR (Zymo Research, Irvine, CA, USA) plant-seed DNA mini-prep extraction kit’s manual with little modification. Briefly, 150 mg of maize grains was ground into fine powder by using commercial blender (BPA free jar, Model: TM-800T). Maize flour was put into ZR BashingBead™ Lysis (Zymo Research, Irvine, CA, USA) tube in which 750 μL of lysis solution was added. Addition of lysis solution was followed by incubating the samples for at least 1 h at room temperature. After incubation time, the tissues were disrupted by shaking in a vortex for 1 min. After vortexing, the next steps were carried out as outlined in the manual. The quality of the DNA yield was tested using an agarose gel electrophoresis. The quantity of DNA extracted was further determined using Quantus™ Fluorometer (Promega, Leiden, The Netherlands). Q-PCR analysis was performed as described by Nicolaisen et al. [53]. The Q-PCR primers were based on the EF1-α sequence, the forward primer used in the reaction mix was 5′-CGTTTCTGCCCTCTCCCA and the reverse primer was 5′-TGCTTGACACGTGACGATGA. The Q-PCR was carried out in a total volume of 12.5 μL consisting of 6.25 μL SYBR Green PCR Master Mix (Applied Biosystems, Foster City, CA, USA), 250 nM of each primer, 0.5 μg/μL bovine serum albumin (BSA) and 2.5 μL template DNA. The Q-PCR was performed on a 7000 Sequence Detection System (SDS) (Applied Bioscience, Mumbai, India) using the following cycling protocol: 2 min at 50 °C; 95 °C 10 min; 40 cycles of 95 °C for 15 s and 62 °C for 1 min followed by dissociation curve analysis at 60 to 95 °C. A no template control and a dilution series of five known template concentrations (1–4 μg/mL–1 μg/mL) were added to establish a standard curve. Results were visualized with the 7000 System Sequence Detection Software (SDS), version 1.2.3 by Applied Biosystems.

### 4.5. Statistical Analysis

Since the condition of normality was not met, the data were brought to the normal distribution by transforming the data using the Arc Sine square root transformation. Analysis of variance (ANOVA) was conducted to determine the effects of crop protection and fertilizer treatments on incidence and severity of infestation of MSB, *Fusarium* ear rot disease symptoms, biomass of *F. verticillioides* and fumonisin content in maize kernels. In case significant differences between treatments were found, a pairwise comparison using a Dunnett T3 test was performed. All statistical tests were conducted at a significance level of α = 0.05. Correlation analysis was performed for symptoms of insect damage, *Fusarium* ear rot, and biomass of *F. verticillioides* and contamination of fumonisins in kernels using the Pearson correlation coefficient. The statistical analysis was done by using the SPSS version 22.0 (SPSS Inc., Chicago, IL, USA). The data are represented as box plots, which provide a graphical view of the median (horizontal line) and quartiles (Q1–Q3, box). The upper whisker is located at the smaller of the maximum *x* value and Q3 + 1.5 × interquartile range, whereas the lower whisker is located at the larger of the smallest *x* value and Q1 − 1.5 × interquartile range. An outlier is defined as a data point that is located outside the whiskers of the boxplot, outside 1.5 times the interquartile range above the upper quartile and below the lower quartile.

## Figures and Tables

**Table 1:** Agro meteorological information of the experimental field during the cropping seasons.

Season		Rainfall (mm)	Dry Days	Rain Days	Temperature (°C)	Humidity (%)
2013	March	278.0	16	15	27.09	58.71
	April	230.4	11	19	23.40	64.93
	May	82.0	19	12	24.57	56.35
	June	16.7	26	5	22.69	44.7
**Total/average**		**607.1**	**72**	**51**	**24.44**	**56.17**
2014	March	182.7	9	21	26.97	57.61
	April	231.0	10	21	25.78	75.88
	May	113.0	14	17	24.17	65.43
	June	24.0	28	3	22.86	58.4
**Total/average**		**550.7**	**61**	**62**	**24.95**	**64.33**

**Table 2:** Effect of growing season on insect pest infestation (Ear and Kernel Injury (EI and KI)), infection of *F. verticillioides* (*Fusarium* Ear and Kernel rot (FER and FKR)), biomass of *F. verticillioides* and contamination with fumonisins (FB1 and FB2). Different letters (superscript) refer to significant differences between growing seasons according to a *t*-test.

Year	EI (%)	KI (%)	FER (%)	FKR (%)	*F. verticillioides* (pg)	FB1 (μg/kg)	FB2 (μg/kg)
2013	19.36 ^a^	24.59 ^a^	11.04 ^b^	18.34 ^a^	2.39 ^b^	4863.25 ^a^	1674.61 ^a^
2014	19.13 ^a^	15.24 ^b^	34.19 ^a^	22.25 ^a^	9.43 ^a^	2407.10 ^b^	626.73 ^b^

**Table 3:** Crop protection products, fertilizer and dosages applied.

Trade Name	Active Ingredient	Dose/ha
Thionex (35EC)	Endosulfan (350 g/L)	2000 mL
Rustal (375EC)	125 g/L triadimenol + 250 g/L tebuconazole	500 mL
Triple super phosphate	Phosphorus (45% P_2_O_5_)	30 kg P_2_O_5_
Urea	Nitrogen (46% N)	40 kg N

## References

[1] Ranum P., Peña-Rosas J.P., Garcia-Casal M.N. (2014). Global maize production, utilization, and consumption. Ann. N. Y. Acad. Sci..

[2] Lukuyu B., Franzel S., Ongadi P., Duncan A. (2011). Livestock feed resources: Current production and management practices in central and northern rift valley provinces of Kenya. Livest. Res. Rural Dev..

[3] Hertel T.W., Golub A.A., Jones A.D., O’Hare M., Plevin R.J., Kammen D.M. (2010). Effects of US maize ethanol on global land use and greenhouse gas emissions: Estimating market-mediated responses. BioScience.

[4] Amare M., Asfaw S., Shiferaw B. (2012). Welfare impacts of maize-pigeonpea intensification in Tanzania. Agric. Econ..

[5] Rowhani P., Lobell D.B., Linderman M., Ramankutty N. (2011). Climate variability and crop production in Tanzania. Agric. For. Meteorol..

[6] Meissle M., Mouron P., Musa T., Bigler F., Pons X., Vasileiadis V., Otto S., Antichi D., Kiss J., Pálinkás Z. (2010). Pests, pesticide use and alternative options in European maize production: Current status and future prospects. J. Appl. Entomol..

[7] Covarelli L., Beccari G., Salvi S. (2011). Infection by mycotoxigenic fungal species and mycotoxin contamination of maize grain in Umbria, central Italy. Food Chem. Toxicol..

[8] Menniti A., Gregori R., Neri F. (2010). Activity of natural compounds on *Fusarium verticillioides* and fumonisin production in stored maize kernels. Int. J. Food Microbiol..

[9] Marin S., Ramos A., Cano-Sancho G., Sanchis V. (2013). Mycotoxins: Occurrence, toxicology, and exposure assessment. Food Chem. Toxicol..

[10] Kamala A., Ortiz J., Kimanya M., Haesaert G., Donoso S., Tiisekwa B., De Meulenaer B. (2015). Multiple mycotoxin co-occurrence in maize grown in three agro-ecological zones of Tanzania. Food Control.

[11] Kimanya M.E., De Meulenaer B., Roberfroid D., Lachat C., Kolsteren P. (2010). Fumonisin exposure through maize in complementary foods is inversely associated with linear growth of infants in Tanzania. Mol. Nutr. Food Res..

[12] Kimanya M.E., De Meulenaer B., Tiisekwa B., Ndomondo-Sigonda M., Devlieghere F., Van Camp J., Kolsteren P. (2008). Co-occurrence of fumonisins with aflatoxins in home-stored maize for human consumption in rural villages of Tanzania. Food Addit. Contam..

[13] Shirima C.P., Kimanya M.E., Kinabo J.L., Routledge M.N., Srey C., Wild C.P., Gong Y.Y. (2013). Dietary exposure to aflatoxin and fumonisin among Tanzanian children as determined using biomarkers of exposure. Mol. Nutr. Food Res..

[14] Venturini G., Assante G., Vercesi A. (2011). *Fusarium verticillioides* contamination patterns in Northern Italian maize during the growing season. Phytopathol. Mediterr..

[15] Murillo-Williams A., Munkvold G. (2008). Systemic infection by Fusarium verticillioides in maize plants grown under three temperature regimes. Plant Dis..

[16] Dohlman E., Buzby J.C. (2003). Mycotoxin Hazards and Regulations Impacts on Food and Animal Feed Crop Trade. International Trade and Food Safety Economic Theory and Case Studies.

[17] Jacobsen B.J., Leslie J.F., Logrieco A.F. (2014). Good agricultural and harvest practices to reduce mycotoxin contamination in wheat in temperate countries. Mycotoxin Reduction in Grain Chains.

[18] Parsons M., Munkvold G. (2010). Associations of planting date, drought stress, and insects with *Fusarium* ear rot and fumonisin B1 contamination in California maize. Food Addit. Contam..

[19] Abbas H.K., Zablotowicz R.M., Weaver M.A., Shier W.T., Bruns H.A., Bellaloui N., Accinelli C., Abel C.A. (2013). Implications of Bt traits on mycotoxin contamination in maize: Overview and recent experimental results in Southern United States. J. Agric. Food Chem..

[20] Folcher L., Delos M., Marengue E., Jarry M., Weissenberger A., Eychenne N., Regnault-Roger C. (2010). Lower mycotoxin levels in Bt maize grain. Agron. Sustain. Dev..

[21] Gatch E., Munkvold G. (2002). Fungal species composition in maize stalks in relation to European corn borer injury and transgenic insect protection. Plant Dis..

[22] Van Rensburg J.B.J. (2007). First report of field resistance by the stem borer, Busseola fusca(Fuller) to Bt-transgenic maize. S. Afr. J. Plant Soil.

[23] Mourice S., Rweyemamu C., Nyambilila A., Tumbo S. (2014). Narrowing Maize Yield Gaps Under Rain-fed conditions in Tanzania: Effect of Small Nitrogen Dose. Tanzan. J. Agric. Sci..

[24] Walters D., Bingham I. (2007). Influence of nutrition on disease development caused by fungal pathogens: Implications for plant disease control. Ann. Appl. Biol..

[25] Munkvold G.P. (2003). Epidemiology of *Fusarium* diseases and their mycotoxins in maize ears. Eur. J. Plant Pathol..

[26] Cao A., Santiago R., Ramos A.J., Souto X.C., Aguín O., Malvar R.A., Butrón A. (2014). Critical environmental and genotypic factors for *Fusarium verticillioides* infection, fungal growth and fumonisin contamination in maize grown in northwestern Spain. Int. J. Food Microbiol..

[27] Samapundo S., Devliehgere F., De Meulenaer B., Debevere J. (2005). Effect of water activity and temperature on growth and the relationship between fumonisin production and the radial growth of *Fusarium verticillioides* and *Fusarium proliferatum* on corn. J. Food Prot..

[28] Mogensen J.M., Nielsen K.F., Samson R.A., Frisvad J.C., Thrane U. (2009). Effect of temperature and water activity on the production of fumonisins by *Aspergillus niger* and different *Fusarium* species. BMC Microbiol..

[29] Popovski S., Celar F.A. (2013). The impact of environmental factors on the infection of cereals with *Fusarium* species and mycotoxin production—A review/Vpliv okoljskih dejavnikov na okuzbo zit z glivami *Fusarium* spp. in tvorbo mikotoksinov-pregledni clanek. Acta Agric. Slov..

[30] Altieri M.A., Nichols C.I. (2003). Soil fertility management and insect pests: Harmonizing soil and plant health in agroecosystems. Soil Tillage Res..

[31] Hoffland E., Jeger M.J., van Beusichem M.L. (2000). Effect of nitrogen supply rate on disease resistance in tomato depends on the pathogen. Plant Soil.

[32] Harrison U.J., Shew H. (2001). Effects of soil pH and nitrogen fertility on the population dynamics of Thielaviopsis basicola. Plant Soil.

[33] Ochola D., Ocimati W., Tinzaara W., Blomme G., Karamura E. (2014). Interactive effects of fertilizer and inoculum concentration on subsequent development of xanthomonas wilt in banana. Aust. J. Agric. Res..

[34] Phelan L.P., Mason J.F., Stinner B.R. (1995). Soil-fertility management and host preference by European corn borer, *Ostrinia mubilalis* (Hubner), on *Zea mays* L.: A comparison of organic and conventional chemical farming. Agric. Ecosyst. Environ..

[35] Ferrigo D., Raiola A., Causin R. (2014). Plant stress and mycotoxin accumulation in maize. Agrochimica.

[36] Reid L.M., Zhu C., Ma B.L. (2001). Crop rotation and nitrogen effects on maize susceptibility to gibberella (*Fusarium graminearum*) ear rot. Plant Soil.

[37] Ariño A., Herrera M., Juan T., Estopañan G., Carramiñana J., Rota C., Herrera A. (2009). Influence of agricultural practices on the contamination of maize by fumonisin mycotoxins. J. Food Prot..

[38] Khattak A.R.A., Khalil S.K. (2009). Plant density and nitrogen effects on maize phenology and grain yield. J. Plant Nutr..

[39] Blandino M., Reyneri A., Vanara F., Pascale M., Haidukowski M., Saporiti M. (2008). Effect of sowing date and insecticide application against European corn borer (*Lepidoptera: Crambidae*) on fumonisin contamination in maize kernels. Crop Prot..

[40] De Curtis F., De Cicco V., Haidukowski M., Pascale M., Somma S., Moretti A. (2011). Effects of agrochemical treatments on the occurrence of *Fusarium* ear rot and fumonisin contamination of maize in Southern Italy. Field Crops Res..

[41] Saladini M.A., Blandino M., Reyneri A., Alma A. (2008). Impact of insecticide treatments on *Ostrinia nubilalis* (Hübner) (*Lepidoptera: Crambidae*) and their influence on the mycotoxin contamination of maize kernels. Pest Manag. Sci..

[42] De La Campa R., Hooker D.C., Miller J.D., Schaafsma A.W., Hammond B.G. (2005). Modeling effects of environment, insect damage, and Bt genotypes on fumonisin accumulation in maize in Argentina and the Philippines. Mycopathologia.

[43] Mazzoni E., Scandolara A., Giorni P., Pietri A., Battilani P. (2011). Field control of *Fusarium* ear rot, *Ostrinia nubilalis* (Hubner), and fumonisins in maize kernels. Pest Manag. Sci..

[44] Alma A., Lessio F., Reyneri A., Blandino M. (2005). Relationships between *Ostrinia nubilalis* (*Lepidoptera: Crambidae*) feeding activity, crop technique and mycotoxin contamination of corn kernel in northwestern Italy. Int. J. Pest Manag..

[45] Miguel Tde A., Bordini J.G., Saito G.H., Andrade C.G., Ono M.A., Hirooka E.Y., Vizoni E., Ono E.Y. (2015). Effect of fungicide on *Fusarium verticillioides* mycelial morphology and fumonisin B(1) production. Braz. J. Microbiol..

[46] Ebelhar S., Hart C., Bradley C. Corn insecticide seed treatment and foliar fungicide effects on corn response to fertilizer nitrogen. Proceedings of the Illinois Fertilizer Conference 2010.

[47] Rossouw J., Van Rensburg J., Van Deventer C. (2002). Breeding for resistance to ear rot of maize, caused by *Stenocarpella maydis* (Berk) Sutton. 1. Evaluation of selection criteria. S. Afr. J. Plant Soil.

[48] Audenaert K., Landschoot S., Vanheule A., Waegeman W., De Baets B., Haesaert G., Thajuddin N. (2011). Impact of Fungicide Timing on the Composition of the Fusarium Head Blight Disease Complex and the Presence of deoxynivalenol (DON) in Wheat. Fungicides-Beneficial and Harmfull Aspects.

[49] Becher R., Hettwer U., Karlovsky P., Deising H.B., Wirsel S.G. (2010). Adaptation of *Fusarium graminearum* to tebuconazole yielded descendants diverging for levels of fitness, fungicide resistance, virulence, and mycotoxin production. Phytopathology.

[50] Sydenham E.W., Shephard G.S., Thiel P.G. (1992). Liquid chromatographic determination of fumonisins B1, B2, and B3 in foods and feeds. J. Agric. Food Chem..

[51] Samapundo S., De Meulenaer B., De Muer N., Debevere J., Devlieghere F. (2006). Influence of experimental parameters on the fluorescence response and recovery of the high–performance liquid chromatography analysis of fumonisin B1. J. Chromatogr. A.

[52] Kamala A., Kimanya M., Haesaert G., Tiisekwa B., Madege R., Degraeve S., Cyprian C., De Meulenaer B. (2016). Local post-harvest practices associated with aflatoxin and fumonisin contamination of maize in three agro ecological zones of Tanzania. Food Addit. Contam. Part A Chem. Anal. Control Expo. Risk Assess..

[53] Nicolaisen M., Supronienè S., Nielsen L.K., Lazzaro I., Spliid N.H., Justesen A.F. (2009). Real-time PCR for quantification of eleven individual *Fusarium* species in cereals. J. Microbiol. Methods.

